# A deep learning-based prediction model of college students’ psychological problem categories for post-epidemic era—Taking college students in Jiangsu Province, China as an example

**DOI:** 10.3389/fpsyg.2022.975493

**Published:** 2022-08-17

**Authors:** Yongheng Liu, Yajing Shen, Zhiyong Cai

**Affiliations:** ^1^Department of Mental Health Education, Nanjing Audit University, Nanjing, China; ^2^Faculty of Statistics and Data Science, Nanjing Audit University, Nanjing, China

**Keywords:** AB-LSTM, psychological problem categories, natural language processing, machine learning, text cluster analysis

## Abstract

For a long time, it takes a lot of time and energy for psychological workers to classify the psychological problems of college students. In order to quickly and efficiently understand the common psychological problems of college students in the region for real-time analysis in the post-epidemic era, 2,000 college students’ psychological problems were selected as research data in the community question section of the “Su Xin” application, a psychological self-help and mutual aid platform for college students in Jiangsu Province. First, word segmentation, removal of stop words, establishment of word vectors, etc. were used for the preprocessing of research data. Secondly, it was divided into 9 common psychological problems by LDA clustering analysis, which also combined with previous researches. Thirdly, the text information was processed into word vectors and transferred to the Attention-Based Bidirectional Long Short-Term Memory Networks (AB-LSTM). The experimental results showed that the proposed model has a higher test accuracy of 78% compared with other models.

## Introduction

As an increasingly popular research question, how to use the data collected on the Internet to automatically assess the mental health of users has been attracting in-depth research by scholars in the fields of computer science, statistics and psychology. The International Conference on Computational Linguistics (ICSL)^1^ has added the Annual Conference on Computational Linguistics and Clinical Psychology (CLPsych) since 2014, which promoted social network-driven automatic assessment of mental health data. However, automated detection and prevention of underlying mental health problems remains a significant challenge due to privacy secrecy and biased research, as well as inadequate models, lack of expertise, or underutilization.

Psychological research should aim at major issues of social and economic development, based on national conditions, pay attention to the frontiers of the world, make full use of scientific research technology, and build a statistical measure of new development patterns. Under the new development pattern, the types of data are complex and diverse, and the amount of data has grown tremendously, which requires new statistical research. Especially in the face of major public health emergencies such as COVID-19, a large amount of complex data was generated in a short period of time. It is even more necessary to combine actual data with new statistical measures, so as to find out the regularity of emergencies in time and conduct people’s health risk assessment. Therefore, strengthening the combination of contemporary psychological research and big data information can carry out effective real-time analysis, which has theoretical and practical guiding significance for formulating countermeasures and suggestions in the future and improving the focus of mental health education in colleges and universities. Although China has taken a series of effective measures to control the spread of COVID-19, the panic and anxiety brought about by the epidemic has an important impact on mental health (Qiu et al., 2020). In particular, college students as a special group has undergone tremendous changes in their learning styles and living conditions under the influence of the epidemic (Kecojevic et al., 2020). Mining and analyzing the big data of college students’ psychological problems, deepening the efficient combination of psychological problems and data information, will help promote the development of precise prevention and intervention for mental health, and allocate resources more scientifically and effectively, thereby promoting the formation of college students’ good mental health (Qiuqing et al., 2022).

We studied the psychological problems of college students under the epidemic prevention and control by means of text information cluster analysis, and constructed an accurate and effective prediction model based on this, which is of great significance to effectively intervene in the psychological problems of college students.

## Literature review

Although the epidemic has been controlled in the more developed countries due the scale of vaccination, small-scale epidemics often fluctuate, which has changed the physiology, psychology and behavior of college students to some extent in the “post-epidemic era” (Ren and Guo, 2020). In particular, the college student group was in a period of mental immaturity and was extremely vulnerable to shocks in the face of external emergencies (Chang et al., 2020). Some studies have found that in the post-epidemic era, the incidence of psychological problems such as depression and anxiety among students is still relatively high (Cheng, 2021). Much of the information extracted from users’ social network data could help mental health professionals assess the severity of users’ psychological problems and better organize the treatment process (Gaur et al., 2019). Therefore, quickly identifying the types of psychological problems of college students is conducive to solving the difficulties they encounter more efficiently in the post-epidemic era.

### Related research on clustering of psychological problems

Since the standards for identifying mental health are relatively abstract, scholars from different countries have different perspectives on the connotation and standards of mental health. For example, American psychologists Maslow and Mittelmann (1941) put forward ten standards of mental health, and pointed out that the standards of mental health are related to age, gender, culture, religious belief, and even country or region. In recent years, as social network platforms have become an inseparable part of people’s lives, the use of network data to evaluate users’ mental health has the advantages of abundant available data, strong timeliness, non-invasive, long-term tracking, wide coverage of evaluation objects, and convenient storage, in which way can effectively overcome the limitations of traditional mental health assessment methods (Ernala et al., 2019). Resnik et al. (2013) used direct topic modeling with LDA to generate interpretable, psychologically relevant “topics” that added value in the predictions of clinical assessments. Acevedo et al. (2021) used an algorithm to classify academic stress levels during COVID-19.

### Related research on psychological problem prediction

Aiming at the prediction of psychological problems in major public health events in the past, Li et al. (2021) studied the mental health status of college students at different stages, and established a variety of scales combined with logistic regression models to explore the factors that affect college students’ psychological problems. They found that during COVID-19 epidemic, acute stress, anxiety and depression were common among college students, and significantly increased in the early stage of the epidemic. Through the experimental simulation of 250 groups of real data, Wang et al. (2019) improved the initial weights and thresholds of the college students’ psychological crisis warning model based on genetic BP neural network by using MATLAB. Ge et al. (2020) used a machine learning approach to conduct a longitudinal survey of college students to understand the prevalence of possible anxiety and possible insomnia, and to identify their risk factors.

To sum up, although some scholars have conducted research on the mental health of college students, they have found that COVID-19 has a greater negative impact on the mental health of college students. However, there is a certain lag in questionnaire research, which may affect the accuracy of related research on college students’ mental health.

The objectives of this research are as follows: (1) Objectively judge the mental health status of college students according to the expressions of the respondents, (2) choose a more effective method to obtain the psychological state of college students by analyzing the text review data, (3) acquire the accurate classification in the face of massive mental health data, in order to solve the psychology problems of college students more efficiently and pertinently, and save medical resources.

## Tools and methods

### Data sources

This study used crawler technology to crawl 1,456 college students’ psychological problems between January 2020 and May 2022 from the “Community Questions” section of the “Suxin” APP, a psychological self-help and mutual assistance platform for college students in Jiangsu Province, as research data. Then, each paragraph in the text comment was divided into a piece of data, and a total of 2000 pieces of data were finally obtained after preprocessing.

### Model introduction

#### Latent Dirichlet allocation

The most commonly used model in text clustering is the vector space model, but the features of this model are extremely sparse, and it is often impossible to measure the similarity between texts by relying on the degree of co-occurrence of words. The Latent Dirichlet Allocation (LDA) (Blei et al., 2003) topic model selected in this paper can perfectly solve this problem and still has excellent performance in short texts. Using LDAvis (Sievert and Shirley, 2014), we visualized topic models to explain topic distributions and their associated terms ordered by probability for further analysis of college students’ psychological problems (see Supplementary Appendix 1).

#### Long-short term memory

Long-short term memory neural network (LSTM) is suitable for dealing with time-varying sequential problems, and the psychotext data to be processed in this study fits this characteristic. The way people usually speak is from front to back, and the next word is built on the logic of the previous word, so using LSTM networks to process mental text data is the most intuitive way. The LSTM’s hidden layers form a closed loop. The weight from LSTM hidden layer to hidden layer is the memory controller of the network, responsible for scheduling memory, and the state of the hidden layer will participate in the next prediction as the memory state at a certain moment (see Supplementary Appendix 2).

### Predictive model of college students’ psychological problem types

#### The proposed attention-based bidirectional long short-term memory networks network model

**FIGURE 1:**
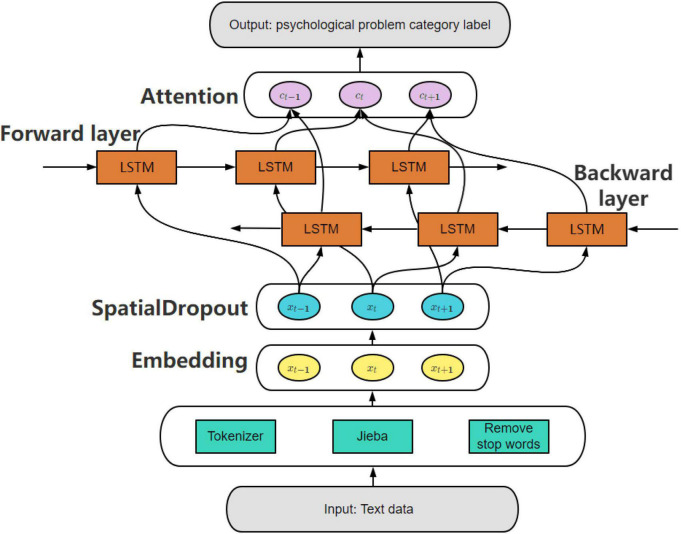
Attention-based bidirectional long short-term memory networks.

The structure of the attention-based bidirectional long short-term memory networks (AB-LSTM) psychological problem type prediction model used in this paper is shown in Figure 1.

**Step1: Text preprocessing.** Data cleaning was performed on the original data, the three parts of Chinese characters, numbers and English letters in the text were preserved, and punctuation marks, emoticons and meaningless characters in the text were deleted. After the cleaning was completed, the jieba Chinese word segmentation database was used for word segmentation, and then the stop words were removed using the stop word list. In order to avoid the interference of some numbers or words, this paper filtered the dictionary obtained after word segmentation, and filtered out more words according to the part of speech of the word segmentation. In order to reduce the complexity of text classification, this paper used keras’ Tokenizer to serialize and vectorize text. First, used the fit_on_texts method of Tokenizer to get the word_index of the mapping relationship between the corresponding words and numbers, and then used texts_to_sequences to get the serialized text data. The padding method was used to make up the same length, and then the Embedding layer that comes with Keras was used for vectorization.

**Step2: Data partitioning.** The original text sample vector obtained above was randomly divided into training set and test set according to the ratio of 9:1.

**Step3:Network structure building.** In this step, the Embedding layer and SpatialDropout1D layer (Salim et al., 2020) are introduced to process the data and output to the Bi-LSTM layer, and the network structure is constructed by combining the attention mechanism.

(1) Embedding layer:Embedding is a way to convert a discrete variable into a continuous vector representation. Embedding can be understood as finding a function or mapping, generating a new spatial expression, and mapping the X space information expressed by the word one-hot to the multi-dimensional space vector of Y. That is, multiply the one-hot encoding matrix by a randomly initialized weight matrix to map it into a new word vector. The mapping relationship is shown in the figure below.


(1) $$\left[0\,\,0\,\,0\,\,1\,\,0\right]\,\left[\begin{array}{cccc} \theta_{11} & \theta_{12} & \theta_{13} & \theta_{14}\\ \theta_{21} & \theta_{22} & \theta_{23} & \theta_{24}\\ \theta_{31} & \theta_{32} & \theta_{33} & \theta_{34}\\ \begin{array}{c} \theta_{41}\\ \theta_{51} \end{array} & \begin{array}{c} \theta_{42}\\ \theta_{52} \end{array} & \begin{array}{c} \theta_{43}\\ \theta_{53} \end{array} & \begin{array}{c} \theta_{44}\\ \theta_{54} \end{array} \end{array}\right]=\left[\theta_{41}\,\begin{array}{ccc} \theta_{42} & \theta_{43} & \theta_{44} \end{array}\right]$$

(2) SpatialDropout1D layer: This paper used the SpatialDropout1 method provided by Ketkar (2017). Each time the layer is updated during training, the input units are randomly set to all zeros at a ratio of 0.2 to a particular latitude, which helps prevent overfitting.

(3) Bi-LSTM layer: Bi-LSTM can utilize the information of early and late sequences (Yu et al., 2020). In this layer, the word vector *w*_*j*_ is combined into a sequence S as the input of Bi-LSTM, and then combined the hidden state *h*_j−1_ generated by the previous layer, to generate the hidden state h_j_ corresponding to each word. The output h of the Bi-LSTM at time t contains forward layer $\vec{h}$ and backward layer $\overset{\leftarrow }{h_{\text{j}}}$, the specific formula is as follows:


(2) $${\vec{h}}_{j}=\mathrm{LSTM}\,\left(\vec{h_{j-1},}\,w_{j}\right)$$

(2)


(3) $${\overset{\leftarrow }{h}}_{j}=\mathrm{LSTM}\,\left(\overset{\leftarrow }{h_{j-1}},w_{j}\right)$$

#### Attention mechanism

Not all words are equally important to a sentence. Some words rich in emotional signals in the sentence, such as adjectives, usually play a decisive role in the emotional attitude of psychological problems. Therefore, the attention mechanism can be used to extract more important textual feature representation*c*_*t*_ (Wang et al., 2016). Within certain time steps, the attention mechanism assigns weights α_*t*,*j*_ to different parts of the text, and the text feature representation ct is calculated as follows.


(4) $$c_{t}=\sum_{j=1}^{n}\alpha_{t,j}\,\left[\vec{h_{j},}\,\overset{\leftarrow }{h_{j}}\right]$$

In this formula, α_*t*,*j*_ is a weight of the feature vector, *h*_*j*_ is the hidden state. In order to calculate α_*t*,*j*_, A layer of feedforward neural network was used to calculate *e_tj_* as a representation of *h*_*j*_. The calculation formula is as follows:


(5) $$e_{\mathrm{tj}}=\delta \,\left(W_{S}\,\left[\vec{h_{j},}\,\overset{\leftarrow }{h_{j}}\right]+b_{s}\right)$$

Where *W*_*s*_ and *b*_*s*_ are the weight matrix and bias, respectively. δ is the non-linear activation function Relu. The calculation of the weight *a*_*t*,*j*_ is as follows:


(6) $$\alpha_{t,j}=\frac{\exp\left(e_{\mathrm{tj}}\right)}{\sum_{j}\exp\left(e_{\mathrm{tj}}\right)}$$

Finally, within certain time steps, *c*_*t*_composes the text feature expression.*c* The final classification result can be expressed as $\hat{y}$.


(7) $$c=\left\{c_{1};c_{2};c_{3};\cdots ;c_{t}\right\}$$

(8) $$\hat{y}=\,\left({\text{w}}_{\text{s}}\,c+{\text{b}}_{s}\right)$$

In this formula,**W**_*s*_ is the weight matrix, and **b**_*s*_ is the bias.

#### Model training

The optimization method selects the Adam algorithm (Kingma and Ba, 2014) to update the model parameters. Because it is a multi-classification problem, the cross-entropy loss is used as the loss function for psychological problem prediction. The expression of the cross entropy loss function is:


(9) $$L=\frac{1}{N}\,\sum_{i}L_{i}=-\frac{1}{N}\,\sum_{i}\sum_{C=1}^{M}y_{\mathrm{ic}}\,\log\left(p_{\mathrm{ic}}\right)$$

Among them, M is the number of psychological problem categories, *y_ic_* is the sign function (0 or 1), if the true category of sample i is equal to c, take 1, otherwise take 0. *p*_ic_ is the predicted probability that the observed sample i belongs to the category c. The specific algorithm is shown in Table 1.

**TABLE 1:** Text content classification algorithm based on AB-LSTM.

1: **Input:** Text data X processed in time series
2: Preprocess the dataset (text data vectorization, data division).
3: Enter the training set and randomly initialize the LTSM model parameters:
4: Build the embedding layer
5: Build the SpatialDropout1D layer
6: Build the LSTM layer
7: Attention (name = “attention_weight”)
8: Build a fully connected layer
10: **Output:** Psychological problem category label

## Experiment and analysis

### Cluster analysis

We used the LDA algorithm to extract 9 topics from the preprocessed documents, and simultaneously calculated the most relevant words and their probabilities under these 9 topics. The input text data was manually checked and summarized according to the given vocabulary, and the nine themes were named: “interpersonal relationship,” “emotional stress,” “academic stress,” “negative life events,” “consult the teacher,” “family relationship,” “mental disease,” “romantic relationship,” “personal growth.”

(1) Interpersonal Relationship (IR): When individuals encounter setbacks in interpersonal relationships and do not know coping strategies, general psychological problems including social fear, interpersonal conflict, interpersonal anxiety, fear of looking at each other, fear of peripheral vision, fear of the opposite sex, fear of telephone calls, communication disorders, fear of rejection, not being rejected, social avoidance, social difficulties, unclear, friendship jealousy, addiction to internet relationships, etc. will arise.

(2) Emotional Stress (ES): The psychological tension reaction or state formed by an individual under the action of emotions such as anxiety or fear. Situational stimuli such as major blows from nature or society, individuals experience emotional stress due to tension when they feel that they are unable to cope.

(3) Academic Stress (AS): In the study life, psychological problems caused by the failure of the election, unsuitable learning method and environment under COVID-19, unsatisfactory academic performance, high pressure, unable to keep up with the school’s training plan and teaching progress and so on.

(4) Negative Life Events (NLE): General psychological problems that an individual has as a result of negative events in his life. Negative life events refer to factors that can negatively affect people’s mental activities, including controllable factors such as quarrels with other people and uncontrollable factors such as natural and man-made disasters.

(5) Consult the Teacher (CT): Inquire about how to use the platform, reply to the teachers on the platform, or have opinions on the teachers of school, and hope that the teachers on the platform can give suggestions, etc.

(6) Family Relationship (FR): Since most college students have just grown up, they still have some rebellious mentality, and sometimes they cannot handle the relationship with their parents well. Such problems include psychological problems caused by conflicts with parents, or not knowing how to communicate with parents.

(7) Mental Disease (MD): Poor psychological tolerance, with serious psychological problems such as depression, bipolar disorder, schizophrenia, anxiety disorder, organic mental disorder and delusional disorder, which are in urgent need of psychological guidance from teachers.

(8) Romantic Relationship (RR): Psychological problems caused by incompatible patterns such as virgin complex, virgin complex, suspicion, emotional trauma, fear of love, emotional conflict, emotional crisis, shadow of lovelorn, mate choice anxiety, rejection of confession, fear of blind date, emotional violence, etc.

(9) Personal Growth (PG): Because individuals do not develop good coping strategies, lack courage and self-confidence, experience bad emotions, and encounter psychological problems caused by setbacks in the process of growth.

**FIGURE 2:**
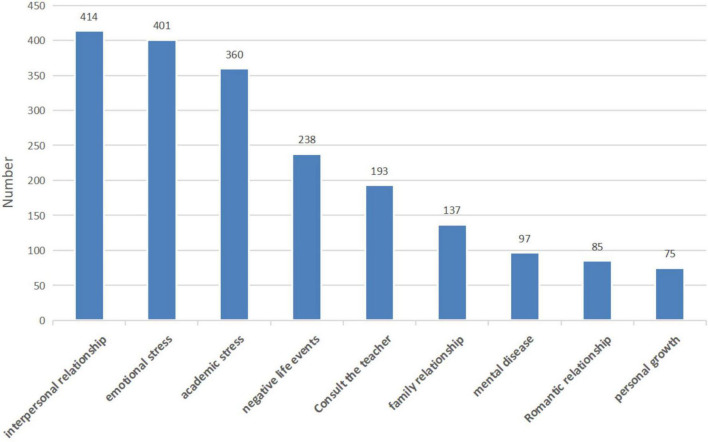
The distribution of psychological problems of college students.

The bar chart is shown in Figure 2. It can be clearly seen that in all the review sample data, there are more problems involved “interpersonal relationship” and “emotional stress” and less problems involved “romantic relationship” and “personal growth.”

### Network structure establishment

Python3 was chosen to built the embedding layer, SpatialDropout1D layer, LSTM layer, and output layer of the AB-LSTM neural network structure. The embedding layer used a vector of length 100 to represent each word. The SpatialDropout1D layer randomly set the ratio of input units to 0.2 at each update during training, which helped prevent overfitting. The LSTM layer contained 100 memory cells. The output layer was a fully connected layer containing 9 categories, and the activation function was a softmax function to calculate the probability of multiple categories. The loss function was the cross entropy loss function, and the optimization algorithm was the Adam algorithm. Used the training data (X_train, Y_train) to fit the model, set the number of network iterations epochs = 15, and the batch size was 32.

### Experimental data set and evaluation index

**FIGURE 3:**
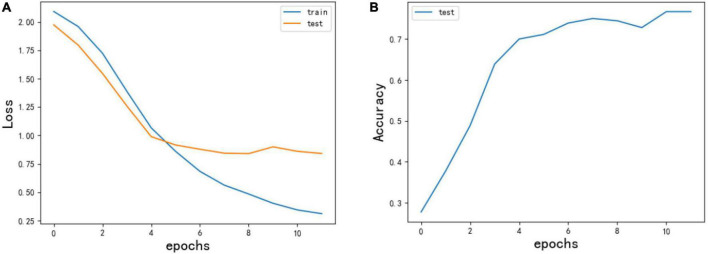
**(A)** Model loss diagram. **(B)** Model accuracy chart.

This research classified the text review data based on the LSTM algorithm, with a total of 2,000 samples, of which the training data accounted for 90% and the test data accounted for 10%. As shown in Figure 3, the left side of the figure was the loss curve of the model, the blue line was the loss of training data, the yellow line was the loss of test data, the abscissa was the number of iterations, and there were 20 iterations in total. When iterating to the 10th round, the loss of the model decreased slowly. Although the loss of training data is still decreasing, the loss of test data was basically stable, and even has an upward trend. The right picture showed the accuracy curve. The model fitting results were good. The highest accuracy rate for the test set was 0.82, and the model was not overfitting.

**FIGURE 4:**
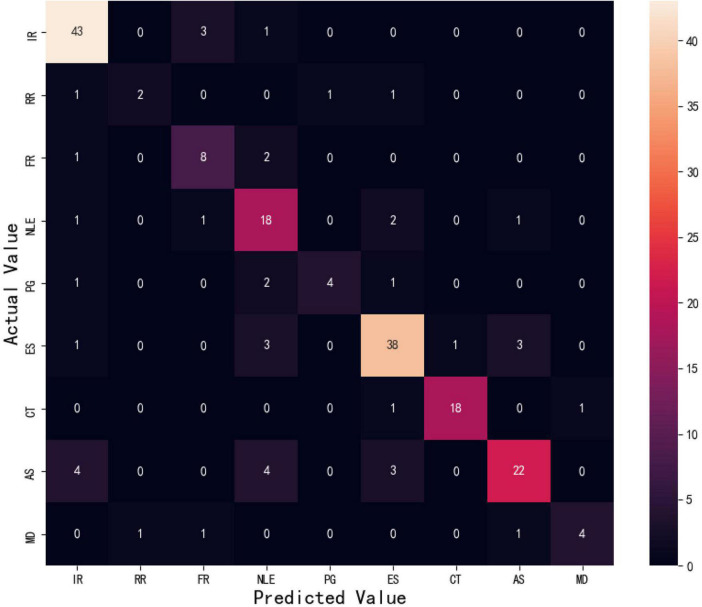
Confusion matrix diagram.

The confusion matrix compared the actual results in the data set with the predicted results in matrix form. The rows of the matrix represented the actual results, and the columns of the matrix represented the prediction results. Figure 4 demonstrates a confusion matrix of the best performing model. Each type of psychological problem is presented in an abbreviated form, such as Ir for Interpersonal relationship. AB-LSTM achieved an accuracy rate of more than 0.6 in each category.

### Model comparison analysis

This paper counted all words (removing duplicates) by counting text words, and then used these words as feature vectors and the number of lines as a dimension. Divided the data set into a test set and a training set, and then used the feature vectorization library to transform the text into feature vectors (converted the text into multi-dimensional feature vectors), and trained the model parameters with the divided training data. Finally, used the trained model to predict the results and compare them with other models.

To verify the effectiveness of the proposed AB-LSTM model in this paper, it is compared with MultinomialNB (Xu et al., 2017) and SVC (Liu et al., 2022). The “Accuracy” for each label is the ratio of the number of correctly classified samples to the total number of samples. Different models have different performances in the test set, and the same model has different prediction accuracy in different topics. In order to compare the accuracy of the classification results of different classification algorithms, the results are shown in Table 2.

**TABLE 2:** Accuracy of different classification algorithms.

Label	MultinomialNB	SVC	AB-LSTM
Interpersonal relationship	0.52	0.62	**0.62**
Emotional stress	0.43	0.78	**0.80**
Academic stress	0.56	0.56	**0.80**
Negative life events	**0.7**	0.58	0.67
Consult the teacher	0.8	**0.86**	0.60
Family relationship	**1**	0.8	0.83
Mental disease	0.5	0.5	**0.95**
Romantic relationship	0	**1**	0.81
Personal growth	**1**	0	0.83

Bold is the highest value.

This table shows the prediction accuracy of three modeling methods for various psychological problems, and the highest value has been bolded. It can be seen from the table that the prediction accuracy of the AB-LSTM model on psychological problems caused by interpersonal relationship, mental disease, emotional stress and other reasons is significantly higher than that of the other two models. It can also maintain a high accuracy rate for psychological problems caused by negative life events, romantic relationship, personal growth and other reasons.

The indicators selected in this paper were: precision rate and recall rate. Calculate the F1 score for measurement. The formula is as follows:


(10) $$\mathrm{Pr}\mathrm{ecision}=\frac{\mathrm{Ture}\,\mathrm{positive}}{\left(\mathrm{True}\,\mathrm{Positive}\,+\mathrm{False}\,\,\mathrm{Positive}\right)}$$

(11) $$\mathrm{Recall}=\frac{\mathrm{Ture}\,\mathrm{positive}}{\left(\mathrm{True}\,\mathrm{Positive}\,+\mathrm{False}\,\mathrm{Negative}\right)}$$

(12) $$\mathrm{F1}\,\mathrm{Score}=\frac{2*\left(\mathrm{Precision}*\text{Recall}\right)}{\left(\mathrm{Precision}+\text{Recall}\right)}$$

In order to exclude the situation that the experimental results were not comparable due to different feature structures, we used the same feature processing except for the different models to ensure the authenticity and reliability of the comparison results. As shown in Table 3, AB-LSTM was superior to other models in precision, recall and F1 score, compared with the MultinomialNB model and the support vector machine model.

**TABLE 3:** Score table of different classification algorithms.

Classification model	Precision	Recall	F1 score
MultinomialNB	0.56	0.53	0.54
SVC	0.66	0.64	0.65
AB-LSTM	**0.78**	**0.76**	**0.77**

Bold is the highest value.

## Conclusion

### Overview

The main research work of this paper was based on the research data of 2000 college students’ psychological problems in the community question section of the “Suxin” APP, a psychological self-help and mutual aid platform for college students in Jiangsu Province. The psychological data obtained in this paper for the first time had no labels, and innovatively introduced LDA clustering into the classification of psychological problems, which changed the previous thinking of simply using artificial methods to study it, and more scientifically clustered the psychological problems of college students. After preprocessing it with machine learning techniques, an improved AB-LSTM was proposed to predict the psychological problems of college students with an average accuracy of 78%. Due to the small sample size, the accuracy of the model has achieved desirable results.

In a one-way LSTM, the model actually only uses the “above” information without considering the “below” information. In the prediction of psychological problems, the information of the entire input sequence needs to be used, so the bidirectional propagation LSTM was adopted, and the attention mechanism was introduced to strengthen the long-distance information in the LSTM.

Although many students’ courses involve mental health, due to the repeated epidemics, students’ needs for psychological counseling have increased significantly. There are convincing evidence that timely counseling can help improve the mental health of college students (Thomas et al., 2014). The experimental results show that interpersonal relationship, emotional stress and academic stress are very common among college students’ psychological problems in the post-epidemic era. Most schools were forced to close down, students rarely participated in group activities, had more contact with roommates, and significantly increased interpersonal problems. At the same time, irritability is easy to appear during the isolation period. Due to the increase in the number of consultations, many students cannot get timely psychological consultation, which makes the emotional pressure continue to increase. As one of the most common emotions students experience in college, academic stress can sometimes have an impact on physical and mental health. Due to the COVID-19 pandemic, colleges and universities around the world have moved away from classrooms to offer virtual teaching methods, creating challenges, adaptations and more stress for students. Through the AB-LSTM model, the prediction accuracy of interpersonal problems is 0.62, the prediction accuracy of emotional stress is 0.8, and the prediction accuracy of academic stress is 0.8, which are better than MultinomialNB model and SVC model.

### Contribution

The current study is one of the attempts to link machine learning theory with the analysis of psychological problems in college students. Using machine learning methods to predict the psychological problems of college students can improve the efficiency of psychological counseling and help managers to further improve the psychological health of college students under the epidemic.

### Limitations

This study has several limitations. First, the model has low prediction accuracy for negative life events and counseling teachers, and cannot identify psychological problems other than these 9 categories. This requires more psychologists to label the data so that the model can identify more types of psychological problems. Secondly, the data are all from Chinese college students, and the results of the study are not universal. At the same time, there is an error in the manual labeling, which interferes with the subsequent model training. Finally, the model can only be used as an auxiliary tool to identify these 9 types of psychological problems, and cannot replace psychological counselors to help college students solve psychological problems.

### Further research

In order to provide better psychological counseling services for college students, further research is needed, mainly focusing on the following issues:

(1) Collect more data on college students from different regions, colleges, and levels, improve samples of psychological problems, and strengthen the prediction model. Universal significance and popularity.

(2) Further optimize the text prediction ability, prediction speed and stability of the algorithm.

(3) After judging the types of college students’ psychological problems, they can give corresponding replies to the problems and reduce the burden of psychological counseling workers.

## Data availability statement

The original contributions presented in this study are included in the article/Supplementary material, further inquiries can be directed to the corresponding author.

## Ethics statement

Ethical review and approval was not required for the study on human participants in accordance with the local legislation and institutional requirements. Written informed consent from the participants or participants legal guardian was not required to participate in this study in accordance with the national legislation and the institutional requirements. Written informed consent was not obtained from the individual(s) for the publication of any potentially identifiable images or data included in this article.

## Author contributions

YL wrote the first draft and revised the manuscript. YS built the framework and revised the manuscript. ZC collected the data. All authors contributed to the article and approved the submitted version.

## Footnotes

1
http://clpsych.org
