# Supplementary material for: A deep learning-based prediction model of college students’ psychological problem categories for post-epidemic era—Taking college students in Jiangsu Province, China as an example
Source: Front Psychol. 2022 Aug 17;13:975493. doi: 10.3389/fpsyg.2022.975493 (PMC9430022; doi:10.3389/fpsyg.2022.975493)
Supplement: Supplementary file 1 [file Data_Sheet_1.docx]

Supplementary Material

# Supplementary Appendix 1

The LDA model is a three-level Bayesian probabilistic graphical model, which consists of three parts: articles, topics and words. The model requires to input a set of articles and hyperparameters during the training phase, where K represents the number of topics in this set of articles. Since it is a hyperparameter, the optimal number of topics $K_{\mathrm{opt}}$ needs to be artificially given. After finishing its training, the LDA model will output the probability distribution on the topic of each article for the articles entered at the beginning. And the probability distribution of each word under the topic will be output for each topic.

Analyzing it from a mathematical point of view, we can understand the LDA model as a three-level Bayesian probability graph model: Assuming that a Dirichlet distribution with a parameter of α randomly produces a multinomial distribution θ, the multinomial distribution θ will randomly give a number z, and another Dirichlet distribution with a parameter of β is randomly distributed K times (K is the number of topics given above) , the randomly obtained K multinomial distributions are named φ, and the final result ω will be randomly obtained according to the zth multinomial distribution φ. Compare the output text information with the original set of text information, the closer the better. Under the premise that the order of words in each article is irrelevant, the probability of disregarding the order can be written as follows:

$\mathbf{P}\left( \boldsymbol{\omega,z,\theta,\varphi;\alpha,\beta} \right)\mathbf{=}\prod_{\mathbf{j=1}}^{\mathbf{M}} \mathbf{P(}\boldsymbol{\theta}_{\mathbf{j}}\boldsymbol{;\alpha)}\prod_{\mathbf{i=1}}^{\mathbf{K}} \mathbf{P(}\boldsymbol{\varphi}_{\mathbf{i}}\boldsymbol{,\beta)}\prod_{\mathbf{t=1}}^{\mathbf{N}} \mathbf{P}\left( \mathbf{z}_{\mathbf{j,t}} | \boldsymbol{\theta}_{\mathbf{j}} \right)\mathbf{P}\left( \boldsymbol{\omega}_{\mathbf{j,t}} | \boldsymbol{\varphi}\mathbf{z}_{\mathbf{j,t}} \right)$ (1)

This is to generate the same probability as the original corpus; where M is the number of text contents and N is a text content with a word.

For the hyperparameter K above, that is, the selection of the optimal number of topics, We uses the coherence score to determine it. Given a text message Z and a set of words $S^{Z}=\left\{ w_{1}^{Z},\cdots,w_{N}^{Z} \right\}$ in the first N text messages Z, the calculation formula of the consistency score is as follows:

$\mathbf{C}\left( \mathbf{Z,}\mathbf{S}^{\mathbf{Z}} \right)\mathbf{=}\sum_{\mathbf{n=2}}^{\mathbf{N}} \sum_{\mathbf{l=1}}^{\mathbf{n-1}} \log\frac{\mathbf{D}_{\mathbf{2}}\left( \mathbf{w}_{\mathbf{n}}^{\mathbf{z}}\mathbf{,}\mathbf{w}_{\mathbf{l}}^{\mathbf{z}} \right)\mathbf{+1}}{\mathbf{D}_{\mathbf{1}}\mathbf{(}\mathbf{w}_{\mathbf{l}}^{\mathbf{z}}\mathbf{)}}$ (2)

In this formula, D_1(w)_ is the number of occurrences of the word w, and D_2(w1, w2)_ is the number of co-occurrences of the words w_1_ and w_2_. A higher consistency score means better interpretability of the text content which means more meaning.

# Supplementary Appendix 2

LSTM neural network, namely long-short term memory neural network, is suitable for dealing with time-varying sequential problems, and the psychotext data to be processed in this study fits this characteristic. Compared with the simple recurrent neural network, the improvement of LSTM neural network is mainly manifested in the following two aspects:

(1) Introduce $\mathbf{c}_{\mathbf{t}}\boldsymbol{\in}\mathbf{R}^{\mathbf{D}}$, which is a new internal state, for the transmission of linear circular information. At the same time, output information to $\mathbf{h}_{\mathbf{t}}\boldsymbol{\in}\mathbf{R}^{\mathbf{D}}$, which is the external state of the hidden layer, and then records the historical information up to the current time t. The internal state c_t_ combines the information of the internal state c_t_ of the previous moment and the information of the candidate state $c_{t}$ of the current moment, and its calculation formula is as follows:

$\mathbf{c}_{\mathbf{t}}\mathbf{=}\mathbf{f}_{\mathbf{t}}\boldsymbol{\odot}\mathbf{c}_{\mathbf{t-1}}\mathbf{+}\mathbf{i}_{\mathbf{t}}\boldsymbol{\odot}\mathbf{c}_{\mathbf{t}}$ (3)

Among them, $\mathbf{i}_{\mathbf{t}}\boldsymbol{\in}\left[ \mathbf{0,1} \right]^{\mathbf{D}}$, $\mathbf{f}_{\mathbf{t}}\boldsymbol{\in}\left[ \mathbf{0,1} \right]^{\mathbf{D}}$ and $\mathbf{o}_{\mathbf{t}}\boldsymbol{\in}\left[ \mathbf{0,1} \right]^{\mathbf{D}}$, are three gates, ⊙ is the product of vector elements, $\mathbf{c}_{\mathbf{t-1}}$ is the internal state (memory unit) at the previous moment, and ${\tilde{\mathbf{c}}}_{\mathbf{t}}\boldsymbol{\in}\mathbf{R}^{\mathbf{D}}$ is the candidate state through the nonlinear function:

${\tilde{\mathbf{c}}}_{\mathbf{t}}\mathbf{=tanh(}\mathbf{W}_{\mathbf{c}}\mathbf{X}_{\mathbf{t}}\mathbf{+}\mathbf{U}_{\mathbf{c}}\mathbf{h}_{\mathbf{t-1}}\mathbf{+}\mathbf{b}_{\mathbf{c}}\mathbf{)}$ (4)

In this formula, X_t_ is the input at the current moment, h_t-1_ is the output at the previous moment, and $\mathbf{W}_{\mathbf{c}}$, $\mathbf{U}_{\mathbf{c}}$, and $\mathbf{b}_{\mathbf{c}}$ are the parameters to be learned.

(2) The information transfer is controlled by introducing three “gates”: input gate $\mathbf{i}_{\mathbf{t}}$, forget gate $\mathbf{f}_{\mathbf{t}}$ and output gate $\mathbf{o}_{\mathbf{t}}$. The calculation formulas for these three gates are as follows:

$\mathbf{i}_{\mathbf{t}}\boldsymbol{=\sigma(}\mathbf{W}_{\mathbf{i}}\mathbf{X}_{\mathbf{t}}\mathbf{+}\mathbf{U}_{\mathbf{i}}\mathbf{h}_{\mathbf{t-1}}\mathbf{+}\mathbf{b}_{\mathbf{i}}\mathbf{)}$ (5)

$\mathbf{f}_{\mathbf{t}}\boldsymbol{=\sigma(}\mathbf{W}_{\mathbf{f}}\mathbf{X}_{\mathbf{t}}\mathbf{+}\mathbf{U}_{\mathbf{f}}\mathbf{h}_{\mathbf{t-1}}\mathbf{+}\mathbf{b}_{\mathbf{f}}\mathbf{)}$ (6)

$\mathbf{o}_{\mathbf{t}}\boldsymbol{=\sigma(}\mathbf{W}_{\mathbf{o}}\mathbf{X}_{\mathbf{t}}\mathbf{+}\mathbf{U}_{\mathbf{o}}\mathbf{h}_{\mathbf{t-1}}\mathbf{+}\mathbf{b}_{\mathbf{o}}\mathbf{)}$ (7)

In these formulas, σ(·) is a logistic function whose output interval is (0,1), $\mathbf{X}_{\mathbf{t}}$ is the input at the current moment, $\mathbf{h}_{\mathbf{t-1}}$ is the external state at the previous moment, $\mathbf{W}_{\mathbf{i}}$, $\mathbf{W}_{\mathbf{f}}$, $\mathbf{W}_{\mathbf{o}}$, $\mathbf{U}_{\mathbf{i}}$, $\mathbf{U}_{\mathbf{f}}$, $\mathbf{U}_{\mathbf{o}}$, $\mathbf{b}_{\mathbf{i}}$, $\mathbf{b}_{\mathbf{f}}$, $\mathbf{b}_{\mathbf{o}}$ are parameters to be learned.

Finally, the output gate $\mathbf{o}_{\mathbf{t}}$ is used to control the transition from the internal state $\mathbf{c}_{\mathbf{t}}$ to the external state $\mathbf{h}_{\mathbf{t}}\boldsymbol{\in}\mathbf{R}^{\mathbf{D}}$ of the hidden layer. The calculation formula is as follows:

$\mathbf{h}_{\mathbf{t}}\mathbf{=}\mathbf{o}_{\mathbf{t}}\mathbf{⨀}\mathbf{tanh}\mathbf{(}\mathbf{c}_{\mathbf{t}}\mathbf{)}$ (8)

In summary, the recurrent unit structure of the LSTM network can be established. That is to say, first use the hidden layer external state $\mathbf{h}_{\mathbf{t-1}}$ at the previous moment and the input $\mathbf{X}_{\mathbf{t}}$ at the current moment to calculate $\mathbf{i}_{\mathbf{t}}$, $\mathbf{f}_{\mathbf{t}}$, $\mathbf{o}_{\mathbf{t}}$ and the candidate state ${\tilde{\mathbf{c}}}_{\mathbf{t}}$ at the current moment. Then calculate the internal state (memory unit) $\mathbf{c}_{\mathbf{t}}$ at the current moment, and finally calculate the external state $\mathbf{h}_{\mathbf{t}}$.

The hidden layers of LSTM are connected to each other to form a closed loop, and the state of each hidden layer is used as the memory at that moment to participate in the next prediction. The processed document space vector $\mathbf{X}_{\mathbf{t}}$ and the hidden state $\mathbf{h}_{\mathbf{t-1}}$ of the previous time step are used as the input of the long short-term memory gate. For multi-classification problems, the fully connected layer calculation result of the sigmoid activation function is used as the output.
